# Supplementary material for: Impact of Sex in Left Atrial Indices for Prognosis of Heart Failure with Preserved Ejection Fraction
Source: J Clin Med. 2022 Oct 7;11(19):5910. doi: 10.3390/jcm11195910 (PMC9571336; doi:10.3390/jcm11195910)
Supplement: Supplementary file 1 [file jcm-11-05910-s001.zip › jcm-1957346-supplementary.pdf]

Table S1. Differences in clinical characteristics before discharge and event rates during the first year in patients with low and high left atrial volume index before enrollment.

	Left atrial volume index		
	≤43 mL/m²	>43 mL/m2	*P value*
Age, years	80 ± 10	82 ± 8	*0.001*
Men, %	49	41	*0.044*
Systolic blood pressure, mmHg	124 ± 19	120 ± 19	*0.011*
Diastolic blood pressure, mmHg	67 ± 12	65 ± 12	*0.055*
Heart rate, bpm	70 ± 15	70 ± 15	*0.458*
Log (NT-proBNP)	2.90 ± 0.54	3.15 ± 0.48	*<0.001*
eGFR, mL/min/1.73m²	46.0 ± 20.4	40.9 ± 17.2	*<0.001*
Atrial fibrillation, %	29	57	*<0.001*
Coronary artery disease, %	16	20	*0.412*
Diabetes mellitus, %	34	33	*0.924*
Dyslipidaemia, %	37	45	*0.035*
Hypertension, %	84	86	*0.303*
Medications			
Beta-blockers, %	47	60	*<0.001*
Calcium-channel blockers, %	53	51	*0.697*
Diuretics, %	77	86	*<0.001*
RAAS inhibitors, %	70	76	*0.108*
Statins, %	29	35	*0.124*
Re-admission for HF, %	26	38	*<0.001*

Values are mean ± standard deviation or percentage. eGFR, estimated glomerular filtration rate; HF, heart failure; NT-proBNP, N-terminal pro-brain natriuretic peptide; RAAS, renin-angiotensin-aldosterone system.

Table S2. Differences in echocardiographic data in patients with low and high left atrail volume index before discharge.

	LAVI		
	Low	High	*P value*
LAD, mm	39 ± 6	47 ± 8	*<0.001*
LAVI, mL/m^2	33 ± 7	69 ± 29	*<0.001*
SV/LAV	0.98 ± 0.37	0.55 ± 0.26	*<0.001*
LVDs, mm	29 ± 6	30 ± 5	*<0.001*
LVDd, mm	45 ± 6	46 ± 6	*<0.001*
LVEF, %	61.1 ± 7.7	60.2 ± 7.9	*0.164*
LVMI, g/m^2	100 ± 29	113 ± 38	*<0.001*
DcT of E wave, sec	0.22 ± 0.06	0.21 ± 0.07	*0.354*
E/e'	13.1 ± 5.5	14.2 ± 6.1	*0.017*
Ed/Ea	0.120 ± 0.055	0.133 ± 0.059	*0.003*

Values are mean ± standard deviation. DcT, deceleration time; E, early transmitral flow velocity; e', onset of early diastolic mitral annular velocity; Ea, arterial elastance; Ed diastolic elastance; LAD, left atrial diameter; LAV, left atrial volume; LAVI, left atrial volume index; LVDs, left ventricularend-systolic dimension; LVDd, left ventricular end-diastolic dimension; LVEF, left ventricular ejection fraction; LVMI, left ventricular mass index; SV, stroke volume.

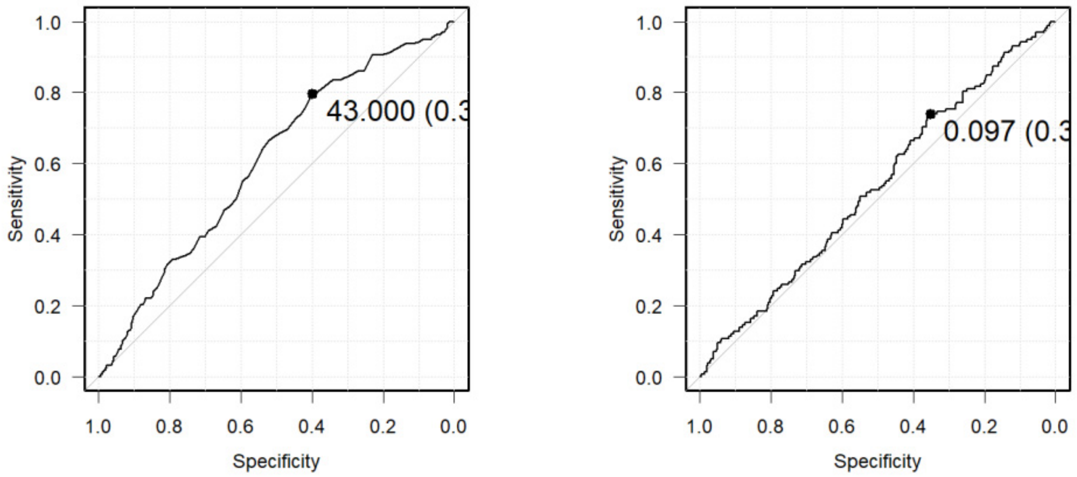

LAVI: AUC 0.606 (95% CI 0.558 - 0.654)

Ed/Ea: AUC 0.533 (95%CI 0.483 - 0.582)

Figure S1: Area under the curve (AUC) calculated by receiver operating characteristic curve analysis in all patients with heart failure with preserved ejection fraction.
